# Chiral Recognition Mechanisms of four β-Blockers by HPLC with Amylose Chiral Stationary Phase

**Published:** 2014

**Authors:** Dongmei Wang, Fang Li, Zhen Jiang, Li Yu, Xingjie Guo

**Affiliations:** School of Pharmacy, Shenyang Pharmaceutical University,China.

**Keywords:** β-blockers, Chiral recognition mechanism, Chiralpak AD-H, HPLC

## Abstract

The high performance liquid chromatography (HPLC) enantioseparation of four β-blocking agents metoprolol, bisoprolol, propranolol and atenolol was performed on amylose tris-(3,5-dimethylphenylcarbamate) chiral stationary phase using *n*-hexane-ethanol-diethylamine (DEA) as the mobile phase and related chiral recognition mechanisms were discussed. Enantiomeric separation of the four β-blockers was a result of more than one type of interaction between solutes and CSP. Besides hydrogen bonding, there was another type interaction that was independent of solvent polarity and responsible for enantiomeric selectivity, such as - interactions. Both the groups close to the chiral centers and the substituent groups on the phenyl rings, which were far away from the chiral centers, could contribute to the good separation. The separations of the four β-blocker enantiomers were all enthalpy driven process. In the range of 293–308K (20–35 ℃), as the temperature increased, the retention as well as the resolution decreased. The molecular size rather than concentration of the alcohol modifiers affected the resolution and retention.

## Introduction

It is well known that enantiomers can exhibit completely different physiological and biological activities, as well as pharmacodynamic and pharmacokinetic characteristics (1), which means enantiomeric forms of a drug can differ in potency and toxicity. The Food and Drug Administration (FDA, U.S.A.), and the regulatory authorities in Europe, China, and Japan have provided guidelines indicating that preferably only the active enantiomer of a chiral drug should be brought to market. Hence, enantiomeric separation has acquired importance in all stages of drug development and the commercialization process. The separation techniques and understanding of related chiral recognition mechanisms are becoming more necessary. Among the chromatographic methods so far developed, HPLC methods with different chiral stationary phases are widely employed for the assays of drug isomers in pharmaceutical preparations and biological fluids (2-5). 

**Figure 1:**
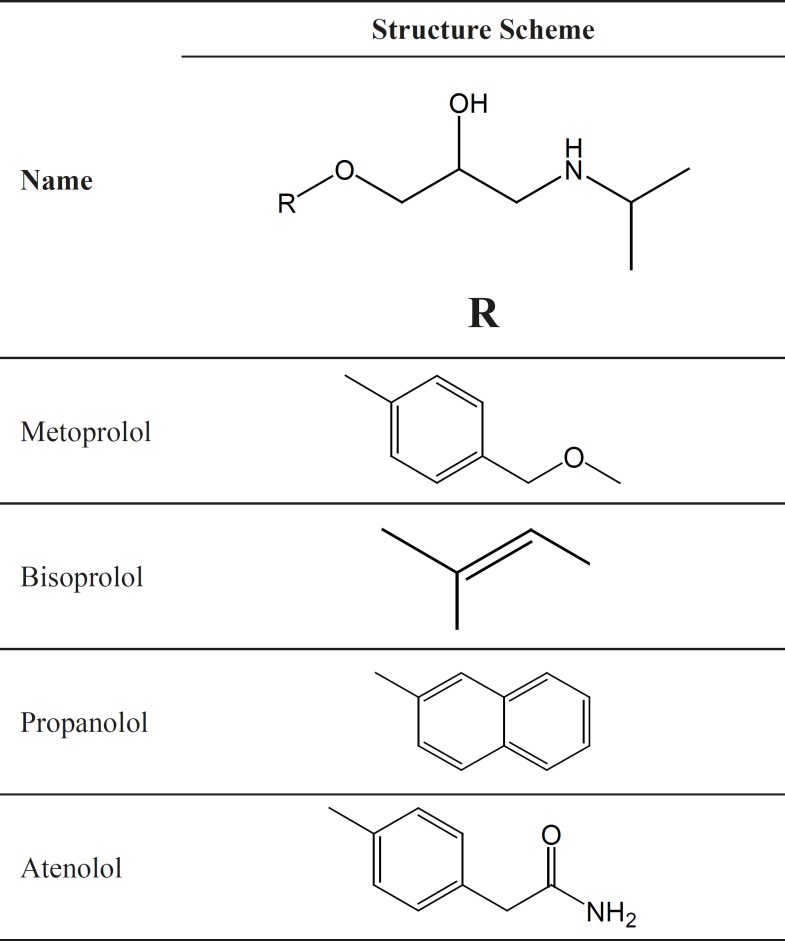
Chemical structures of the four β-blockers

Atenolol, metoprolol, propranolol and bisoprolol are β-blocking agents that can block adrenergic stimuli, and are responsible for the stimulation of heart and inhibition of several types of smooth muscles. That is why these four agents are used for the treatment of various disorders associated with the circulatory system such as hypertension, anginapectoris, cardiac arrhythmias, glaucoma, supraventricular and ventricular arrhythmias. All of these drugs have one chiral centre in their structures (Figure 1) and are marketed as racemic mixtures. However, it has been demonstrated that the (S)-isomers have much greater affinity (50–500 folds) for binding to the β-adrenergic receptors than the antipodes (6, 7). It therefore is of great importance to develop a rapid and selective enantiomeric separation method for the assays of these chiral drug enantiomers.

A literature survey shows that enantiomeric resolution of β-blockers were carried out by thin layer chromatography method (TLC) (8), electromigration techniques (9, 10) and liduid chromatography method (LC) on cyclodextrin bonded column (11, 12) or Pirkle–1J column (13). Since the 1980s, polysaccharide derivatives have attracted particular interests in the development of CSPs and are currently the most popular CSPs (14, 15). Of the derivatives, the amylose derivatized with tris-(3, 5-dimethylphenylcarbamate) was a better type used in chiral recognition. However, the exact mechanism of chiral separations is not completely understood. Imran Ali *et al.* concluded that the chiral resolution was due to the overall combination of all types of bondings. No single bonding was capable for the enantiomeric resolution of the reported molecules (16). Thus, not only the steric but also the substitutes of a certain chiral compound and of the CSP have to be taken into consideration to elucidate chiral recognition mechanisms (17). One way to elucidate the complex retention mechanism is to examine the temperature dependence of retention and enantioselectivity. Insight can then be obtained from van’t Hoff plots, *i.e*., the natural logarithm of the retention factor or enantioselectivity versus the reciprocal of absolute temperature (18). Another way to study the chiral recognition mechanisms is to investigate and understand the effects of mobile-phase modifiers on the column selectivity. Based on speculation or spectroscopic evidence, the researchers believed that the mobile-phase modifiers in the mobile phase not only competes for chiral bonding sites with chiral solutes, but can also alter the steric environment of the chiral grooves on the CSP by binding to achiral sites at the groove or close to (19, 20). 

**Figure 2:**
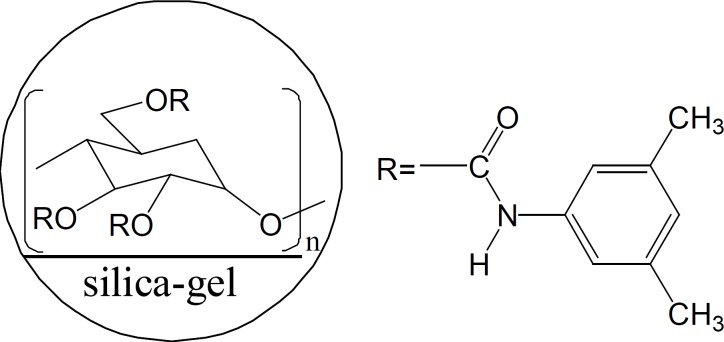
Structure of polysaccharide CSP, Chiralpak AD-H

In this study, we chose four β-blocking agents with similar chiral environment and different achiral group as analytes to study the effect of functional groups on the interaction between solutes and the CSP. The effects of both temperature and mobile phase modiﬁers on the separation of β-blocking agents were also discussed. Overall, the chiral recognition mechanism of four β-blockers by HPLC with amylose chiral stationary phase was revealed. Meanwhile, simple and efficient HPLC method using Chiralpak AD-H as chiral stationary phase (Figure 2) was developed for direct enantioseparation of four β-blocking agents metoprolol, bisoprolol, propranolol and atenolol.

## Experiment

*Reagents and chemicals*

The reference compounds atenolol, metoprolol, propranolol and bisoprolol (purity 98%) were purchased from National Institute for the Pharmaceutical and Biology Products. Ethanol, *n*-propanol, *n*-butanol, iso-propanol and *n*-hexane were of HPLC grade and obtained from Corncord Tech (Tianjin, China). All the other reagents were of analytical reagent grade. 

*Instruments*

The commercially obtained Chiralpak AD-H column was used for chromatographic separations (250 mm × 4.6 mm *i.d*., 5 μm particle size, Daicel Chemical Industries, *Ltd*., Japan). The liquid chromatography system consisted of a LC-10A system (shimadzu, Kyoto, Japan) equipped with a LC-10AD pump, a fixed injection-loop of 20 μL and a SPD-10A UV-vis detector. Data processing was performed using the Anastar software (Tian Jin, China).

*Chromatographic conditions*

The optimized mobile phase composition was n-hexane / EtOH / DEA(75/25/0.1, v/v/v) for metoprolol enantiomers, *n*-hexane / EtOH / DEA (83/17/0.1, v/v/v) for propranolol enantiomers, *n*-hexane / EtOH / DEA(88/12/0.1, v/v/v) for bisoprolol enantiomers and *n*-hexane / EtOH / DEA (80/20/0.1, v/v/v) for atenolol enantiomers respectively. The prepared mobile phase was filtered through a 0.45 µm filter and then degassed before the application. The flow-rate was 0.6 mL/min and the column temperature was set at 20 ℃. The optimum detection wavelength was 270 nm.

## Results and Discussion

*Effect of the organic modifier type and concentration*

Base or acid is commonly used as mobile phase additive in the polysaccharide CSPs in order to obtain better resolution or improve the peak shape (21). Diethylamine (DEA) was chosen in this study to reduce peak tailing by shielding the residual silanol groups of CSP. When 0.1% of DEA was added into the mobile phase, the peak shape did improve and the analysis time was shortened (within 35 min). Higher concentrations of DEA were also evaluated, however, the retention factors and resolution remained almost the same as 0.1%. Therefore, 0.1% of DEA was added into mobile phase for the separation of the enantioseparations.

Several kinds of mobile phase compositions were investigated by changing the nature and the percentage of the alcohol organic modifiers (Table 1). Baseline separation (R_s_ > 1.5) was obtained for all compounds in *n*-hexane/ethanol system. The replacement of ethanol by iso-propanol, the most common organic modifier used on AD-H column (22), leaded to decrease of enantiomeric separation factor of metoprolol and bisoprolol with 1.18<<1.29, while, propranolol and atenolol with =1. When *n*-propanol was used as modifier, only metoprolol and bisoprolol obtained separation with 1.43<R_s_<2.40 and 1.19<<1.22, propanolol was partially resolved with 0.60<R_s_<1.33 and =1.13. Of the four alcohol modifiers used, *n*-butanol showed the lowest selectivity: only metoprolol and bisoprolol could be partially enantioseparated. This phenomenon could be explained by the difference in the steric bulkiness around the hydroxyl moiety of the mobile phase modifiers (23). Amylose based chiral stationary phase is believed to have a four-fold helical backbone (24, 25), the lower alcohols could insert into well deﬁned grooves of the CSP more easily than bulkier alcohols to form more stable diastereomeric complexes with the enantiomers and cause higher resolution with selectivity. 

**Table 1:** The effect of alcohol on selectivity and resolution of β-blocker enantiomers on Chiralpak AD-H column

**Compound **	**Eluent**	**k**_1_	***α***	***R***_s_
Propanolol	A(80:20) A(83:17) A(85:15) A(88:12) B(75:25) B(80:20) B(85:15) B(90:10) C(80:20) C(85:15) C(90:10) D(80:20) D(85:15) D(90:10)	0.60 0.66 0.79 0.97 0.55 0.66 0.84 1.36 0.90 0.96 1.38 0.67 0.92 1.40	1.86 1.85 1.87 1.87 1.00 1.00 1.00 1.00 1.13 1.13 1.14 1.00 1.00 1.00	3.17 4.01 4.12 4.94 n.r. n.r. n.r. n.r. 0.60 0.96 1.33 n.r. n.r. n.r.
**(1)**				
**Compound **	**Eluent**	**k**_1_	***α***	***R***_s_
Metoprolol	A(60:40) A(65:35) A(70:30) A(75:25) B(75:25) B(80:20) B(85:15) B(90:10) C(80:20) C(85:15) C(90:10) D(80:20) D(85:15) D(90:10)	0.52 0.65 0.88 0.98 0.68 0.86 1.11 1.91 1.16 1.33 2.04 1.18 1.63 2.93	1.64 1.58 1.58 1.55 1.28 1.29 1.29 1.28 1.21 1.22 1.22 1.08 1.06 1.06	2.18 2.19 2.97 2.91 0.98 1.98 2.34 3.10 1.75 1.92 2.40 <0.5 <0.5 0.73
**(2)**				
**Compound **	**Eluent**	**k**_1_	***α***	***R***_s_
Bisoprolol	A(82:18) A(85:15) A(88:12) A(90:10) B(75:25) B(80:20) B(85:15) B(90:10) C(80:20) C(85:15) C(90:10) D(80:20) D(85:15) D(90:10)	0.83 1.23 1.69 1.93 0.51 0.68 0.91 1.79 0.96 1.13 2.24 0.99 1.41 2.51	1.59 1.55 1.62 1.59 1.25 1.26 1.25 1.18 1.19 1.21 1.21 1.08 1.51 1.07	2.43 2.43 3.61 4.59 0.50 1.41 1.63 2.40 1.43 1.70 2.30 <0.5 <0.5 0.70
**(3)**				
**Compound **	**Eluent**	**k**_1_	***α***	***R***_s_
Atenolol	A(70:30) A(75:25) A(80:20) A(82:18) B(75:25) B(80:20) B(85:15) C(80:20) C(85:15) C(90:10) D(80:20)	1.15 2.06 3.22 5.00 1.15 1.77 3.00 2.45 4.05 9.94 3.02	1.25 1.26 1.26 1.28 1.00 1.00 1.00 1.00 1.00 1.00 1.00	1.13 1.61 2.07 2.11 n.r. n.r. n.r. n.r. n.r. n.r. n.r.
**(4)**				

n.r: Not resolved. All mobile phase contained 0.1% DEA. The flow-rate was 0.6 mL·min^-1^. The temperature was 20 ℃. The mobile phase were referenced as A, B, C or D for mixtures of hexane (v/v) and ethanol, iso-propanol, *n*-propanol or *n*-butanol as alcohol modifiers, respectively. Detection wavelengths are 270 nm for all compounds.

**Figure 3:**
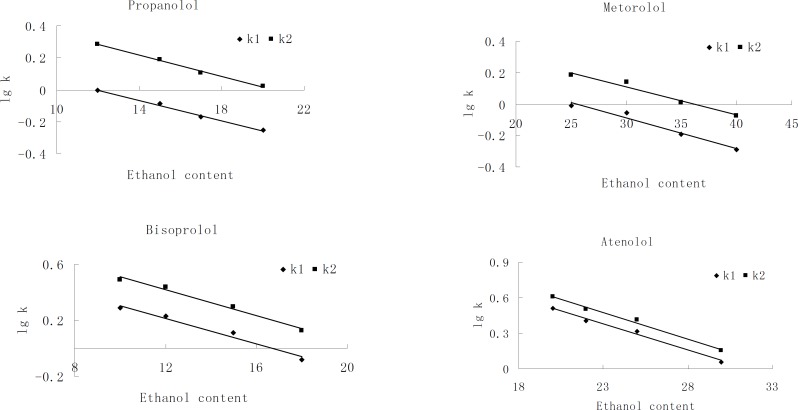
Dependence of lgk for the four compounds on ethanol content

There was a linear relationship between the alcohol modifiers content in the mobile phase and *lgk *of each enantionmer. Interestingly, the enantiomeric separation factor (*α*) was essentially unchanged over the entire range of the alcohol modifiers concentration. The influence of ethanol content on the retention factors (*k*) of each enantiomer was studied in detail. Plots of lg*k* versus ethanol concentration were made as shown in Figure 3. The result suggests that, at a constant temperature, the conformation of the polymeric phase, the selective adsorption sites, and the selector associate are not affected by alcohol modifier concentration. Hence, enantiomeric separation is a result of interaction between β-blocking agents and CSP. We considered that, besides hydrogen bonding, there is another type of interaction that is independent of solvent polarity and responsible for enantiomeric selectivity, such as - interactions.

**Figure 4:**
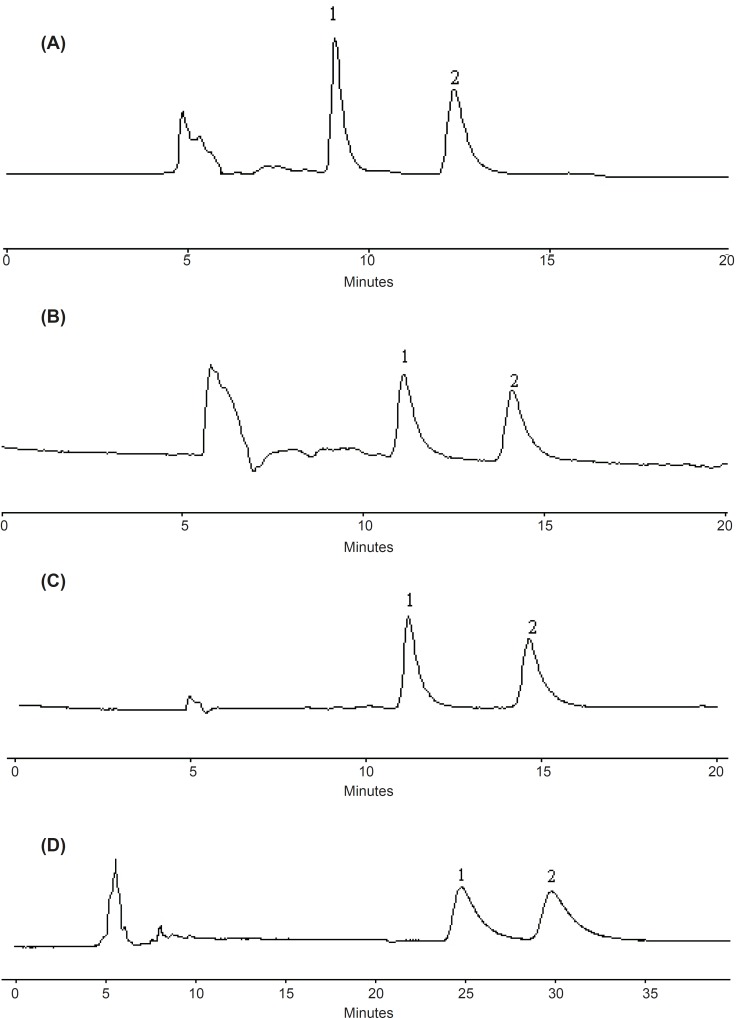
Chromatograms of chiral separation of four β-adrenergic blockers enantiomers.

Considering a compromise between resolution and retention, 25%, 17%, 12% and 20% of ethanol in *n*-hexane were found to be optimum composition of the mobile phase for metoprolol, propranolol, bisoprolol and atenolol, respectively, with the flow-rate of 0.6 mL/min and column temperature at 20 ℃. The representative chromatograms are shown in Figure 4.

(a)Propanolol enantiomers (b)Metoprolol enantiomers (c)Bisoprolol enantiomers (d)Atenolol enantiomer .The flow-rate was 0.6 mL/min and the column temperature was 20 ℃. The optimum detection wavelength was set at 270 nm.

*Effect of temperature on enantiomeric separation*

The effect of column temperature on resolution, retention factors and separation factors of four β-blocker enantiomers was studied. In the range of 293–308 K (20–35 ℃), as the temperature increased, the retention as well as the resolution decreased (data not shown). According to the van’t Hoffs equation, the retention factors are related to temperature (26). 

Since 

*△△* *G° =* *△△* *H* *－T△△S* and *△G°=－RT* ln (*k*)

ln(*k’*)_1_ =*－△H°*_1_*/RT*﹢*△S°*_1_*/R*

ln(*k’*)_2_=*－△H°*_2_*/RT*﹢*△S°*_2 _*/R*

Where *k *is retention factors, R is the gas constant, T is the absolute temperature, *△H° *and *△S° *represent the enthalpy and entropy differences of the enantiomers interacted with the stationary phase, respectively. Van’t Hoff plots were drawn with natural logarithm of retention factors (ln*k*) versus inverted temperature (1/*T*) for the enantiomers. The linear character suggested that the conformation of the CSP did not change substantially within the range of experimental temperature. *△H° *and *△S° *were obtained from slope and intercept of the straight lines, respectively. The enthalpy change (*△△H°*), entropy change (*△△S°*) and Gibb’s free energy change (*△△G°*) were recorded in Table 2. The estimated values for *△H°* and *△S°* for all solutes are found to be negative. These values indicate that solute transfer from the mobile to stationary phase is enthalpically favorable but entropically unfavorable (18). The values of *△△H°* and *△△S°* are negative for the enantioseparation of enantiomers indicating that the enantioseparation of β-blocker enantiomers is an enthalpy driven process and the separation factor (*α*) decreased with increasing temperature. Thus, the lower temperature, the better separation result could be obtained. So the column temperature was set at 20 ℃.

**Table 2:** Thermodynamic data calculated from the Van’t Hoff plots of β-blockers enantiomers.

	***△*** ***H°***_1_ ***△H°***_2_ **(kJ·mol**^-1^**)**		***△△*** ***H°*** **(kJ·mol** ^-1^ **)**	***△*** ***S°***_1_ ***△S°***_2_ **(J·mol**^-1^**·K**^-1^**)**		***△△*** ***S°*** **(J·mol** ^-1^ **·K** ^-1^ **)**	***△△*** ***G°*** **(kJ·mol** ^-1^ **,T=** **20** **℃** **）**
Bisoprolol Proranolol Atenolol Metoprolol	-2.64±0.10 -2.54±0.11 -12.15±0.50 -9.23±0.50	-3.74±0.22 -3.67±0.13 -10.32±0.40 -11.61±0.44	-1.10±0.08 -1.13±0.11 -1.82±0.24 -2.38±0.16	-28.76±0.10 -9.91±0.25 -24.51±0.18 -31.53±0.15	-37.85±0.67 -8.95±0.18 -28.78±0.30 -35.86±0.30	-9.09±0.15 -0.96±0.07 -4.28±0.11 -4.34±0.13	-1.57±0.08 -1.41±0.04 -0.57±0.04 -1.11±0.04

*Effect of Analyte structure*

Okamoto Y *et al.* concluded that the most important interactions between the analyte and the CSP are hydrogen bonding, dipole–dipole interactions, and － interactions, together with the rigid structure (cellulose-based CSP) or helical structure (amylose-based CSP) of the chiral polymer bound to the support (27). In our study, the four β-blockers have the similar chiral environment (Figure 1) and the OH group, NH group and oxygen atom on each chiral center of all the solutes are available functional groups for forming hydrogen bonding with the C = O and NH group of the CSP. While, the four solutes had different chiral separation, which indicates that achiral part namely R part probably performs a main role in chiral separation. The － interaction between the substituted phenyl rings in R part and the CSP may be important. Hence, both the groups close to the chiral centers and the substituent groups on the phenyl rings, which are far away from the chiral centers, could contribute to the good separation result. 

Except propanolol, the substituent groups on phenyl ring of the compounds can be regarded as aldyl groups, so the electronic effect of phenyl ring could be ignored. Atenolol had the strongest retention on the CSP with 1.15<*k*_1_<9.94, which may be explained that amide group in R part of atenolol can also form hydrogen bonding and dipole–dipole interactions with CSP. At the same time, the amide group could compete with the groups connected to the chiral carbon for the bonding sites on CSP, which cause atenolol to have the lower stereoselectie interaction (the lowest α and R_s_). Propanolol had the largest *α* in ethanol as organic modifier. A possible reason is that the naphthalene ring of propanolol could form stronger － interactions with CSP. Metoprolol and bisoprolol with similarly R part had similar *α *in different organic modifiers. 

## Conclusions

The utility of AD-H column for the efficient chiral separation of β-blocker metoprolol, bisoprolol, propranolol and atenolol was demonstrated. The effects of temperature, alcohol modiﬁers in mobile phase and structure of the analytes on the separation of β-blocking agents were discussed. The enantioseparation of the four β-blocker enantiomers is an enthalpy driven process and the separation factor (*α*) decreased with increasing temperature. The different steric bulkiness around the hydroxyl moiety of the four alcohol modifiers results in the different chiral resolution. At a constant temperature, the conformation of the polymeric phase, the selective adsorption sites, and the selector associate are not affected by alcohol modifier concentration. Both the groups close to the chiral centers and the substituent groups far away from the chiral centers on the phenyl rings could contribute to the good separation result. Besides hydrogen bonding, there is dominant - interaction between β-blocking agents and CSP. 

Hexane/Ethanol/DEA systems is the most optimal mobile phase for the enantiomeric separation of the β-blockers with R_S_>1.5 and *α* >1.2. The methods developed in this study are adequate for the separation of β-blockers metoprolol, bisoprolol, propranolol and atenolol enantiomers, or further pharmacological investigations.
